# Reduction in Emergency Department Presentations in a Regional Health System during the Covid-19 Pandemic

**DOI:** 10.5811/westjem.2020.10.49759

**Published:** 2021-06-29

**Authors:** Edana Mann, Daniel Swedien, Jonathan Hansen, Susan Peterson, Mustapha Saheed, Eili Klein, Ajit Munjuluru, James Scheulen, Gabor Kelen

**Affiliations:** * Johns Hopkins University School of Medicine, Department of Emergency Medicine, Baltimore, Maryland; † Johns Hopkins Office of Critical Care Event Preparedness and Response, Department of Emergency Medicine, Baltimore, Maryland; ‡ Johns Hopkins University School of Medicine, Armstrong Institute for Patient Safety and Quality, Baltimore, Maryland

## Abstract

**Introduction:**

Nationally, there has been more than a 40% decrease in Emergency Department (ED) patient volume during the coronavirus disease 2019 (Covid-19) crisis, with reports of decreases in presentations of time-sensitive acute illnesses. We analyzed ED clinical presentations in a Maryland/District of Columbia regional hospital system while health mitigation measures were instituted.

**Methods:**

We conducted a retrospective observational cohort study of all adult ED patients presenting to five Johns Hopkins Health System (JHHS) hospitals comparing visits from March 16 through May 15, in 2019 and 2020. We analyzed de-identified demographic information, clinical conditions, and ICD-10 diagnosis codes for year-over-year comparisons.

**Results:**

There were 36.7% fewer JHHS ED visits in 2020 compared to 2019 (43,088 vs. 27,293, P<.001). Patients 75+ had the greatest decline in visits (−44.00%, P<.001). Both genders had significant decreases in volume (−41.9%, P<.001 females vs −30.6%, P<.001 males). Influenza like illness (ILI) symptoms increased year-over-year including fever (640 to 1253, 95.8%, P<.001) and shortness of breath (2504 to 2726, 8.9%, P=.002). ICD-10 diagnoses for a number of time-sensitive illnesses decreased including deep vein thrombosis (101 to 39, −61%, P<.001), acute myocardial infarction (157 to 105, −33%, P=.002), gastrointestinal bleeding (290 to 179, −38.3%, P<.001), and strokes (284 to 234, −17.6%, P=0.03).

**Conclusion:**

ED visits declined significantly among JHHS hospitals despite offsetting increases in ILI complaints. Decreases in presentations of time-sensitive illnesses were of particular concern. Efforts should be taken to inform patients that EDs are safe, otherwise preventable morbidity and mortality will remain a problem.

## INTRODUCTION

According to the Johns Hopkins Coronavirus Resource Center, the United States experienced over 1.6 million coronavirus disease 2019 (COVID-19) infections and more than 125,000 deaths as of June 29, 2020.^1^ The pandemic has created a public health emergency due to a combination of factors including high transmissibility, asymptomatic infectious carriers, and without widespread testing, a difficult-to-calculate infection fatality rate (IFR).^2^

Nationally, there has been a greater than 40% decrease in emergency department (ED) patient volume during this crisis.^3^–^5^ Reports have suggested that certain time sensitive presentations requiring immediate medical attention, have decreased as well.^6^–^8^ Investigators in Italy reported an increase in out-of-hospital-cardiac arrests (OHCA) that appears strongly correlated with an increasing incidence of COVID-19 in the community.^9^ Similarly, in California, EMS reported sudden increases in out of hospital cardiac arrests (OHCA) in COVID-19 negative patients, as well as patients arriving too late to receive tissue plasminogen activator for ischemic strokes.^4^ Another Italian report highlights a significant decrease in ischemic stroke presentations at hospitals.^10^ While each of these is a concern in and of itself, there has been few detailed analyses characterizing the variance in the multiplicity of patient conditions associated with the ED volume loss.

We sought to determine and characterize the change in ED presentations during a period while public health mitigation orders were in effect in Maryland and D.C. (March 16, 2020 school closures to May 15, 2020 non-essential businesses reopen in Maryland; March 24, 2020 non-essential business closures to May 29, 2020 Phase One re-opening in D.C.).^11^–^15^ We compared patient volumes, demographics and clinical conditions from March 16th through May 15, 2020 to corresponding dates in 2019 for five regionally dispersed EDs in our health system.

## METHODS

### Study Design and Setting

**Figure 1:**
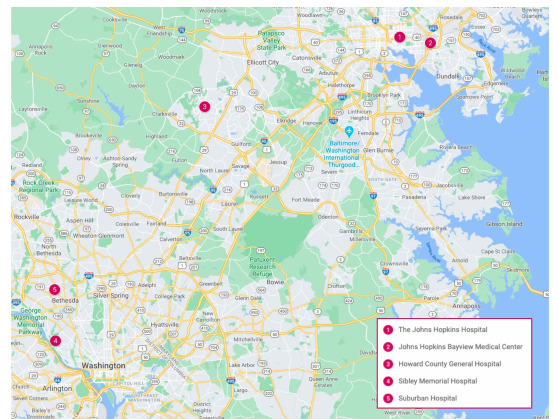
Johns Hopkins Health System (JHHS). Hospital Emergency Departments in Red. Adapted from google map.^16^

We conducted a multi-center retrospective observational cohort study of all registered adult ED patients presenting to any of our five Johns Hopkins Health System hospitals in the mid-Atlantic region. Four of the hospitals are in Maryland and one is in the District of Columbia. The regional hospitals include: a large inner-city academic medical center, an urban community-oriented teaching affiliate, and three community-based non-teaching hospitals. (Figure 1) The study was accepted by the Johns Hopkins Institutional Review Board.

### Study Population

All patients aged 15 years or older who presented to each of our five health-system adult EDs from March 16 through May 15, in 2019 and 2020, respectively, were included. Patients who registered but left without being seen were included. Patients younger than 15 years were excluded from the data set.

### Data Collection, Outcomes, and Analysis

To identify historical patterns, patient volumes for the 2-month period of interest were obtained for the years 2016–2020 for all sites. All data were abstracted from the EPIC electronic medical records (EMR) of our institutions by an experienced data analyst. For 2019 and 2020, we collected de-identified demographic information such as age, sex, race, ethnicity, as well as presenting chief complaints, dispositions, triage assessments (Emergency Severity Index, HopScore), and primary ICD-10 codes. HopScore is an outcomes-based emergency triage system.^17^

**Population Health Research Capsule**

What do we already know about this issue?

*During the first wave of the coronavirus disease 2019 (COVID-19) pandemic in the US, there were dramatic decreases in the number of patients presenting to emergency departments*.

What was the research question?

*Were there any changes in the clinical conditions presenting to a regional health system during the Covid-19 pandemic?*

What was the major finding of the study?

*At the onset of the Covid-19 pandemic, many patients with critical and even fatal illnesses failed to seek emergency care*.

How does this improve population health?

*This study highlights the need for widespread communication to the public regarding the safety of emergency departments and the serious implications of avoiding emergency care*.

Chief complaints with fewer than 15 occurrences were compiled into the “General” category. This included the autoimmune, cancer, dialysis, endocrine metabolic, mass, and transplant categories. Trends over time in visits were calculated for each hospital. For both study periods (2019 and 2020), differences in results across all JHHS EDs was judged as relatively minor. Accordingly, aggregated data was used to identify generalizable trends and to make specific year-over-year comparisons.

Decreases from year to year were calculated both as absolute reductions and percentage changes. As the rate of visits to EDs typically follows a Poisson distribution we used the two-sided Poisson test of two means to assess whether the rate of visits over the two-month study period in 2020 was statistically discernable from 2019.^18^–^20^

## RESULTS

**Figure 2:**
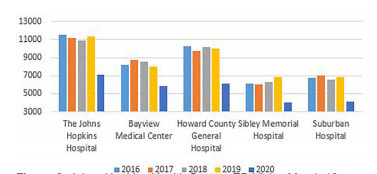
Johns Hopkins Health System ED Visits, March 16 – May 15, 2016 – 2020, respectively.

Patient volumes from 2016 to 2019 averaged 42,775 and no year deviated by more than 1.5% over the corresponding two-month study timeframe in any other year, until 2020. In 2019, there were in aggregate 43,088 visits in all five EDs, and 27,293 for the same study time period in 2020, representing a 37% decrease (P<.001). Decreases across all five EDs ranged from 27.7% to 40.3%. (Figure 2). Similar decreases were seen across almost all demographic groups. There was a decline in visits across all age groups, with the largest decrease in those over the age of 75 (−44.00%, P<.001). During the same time period, there was a greater decrease in patients identifying as females (−41.9%, P<.001) than males (−30.6%, P<.001). There were decreases in all self-identified racial groups who had more than 30 visits. There was no appreciable difference in visits amongst those identifying as Hispanic or Latinx 0.7% (P=0.79) compared to significant declines amongst other self-identified ethnicities (Table 1).

Most clinical conditions, with the exception of pulmonary, influenza-like illness (ILI) and penetrating trauma decreased. Conditions decreasing 60% or greater year-over-year were allergy (311 to 108, −65.3%, P<.001), back pain (1192 to 435, −63.5%, P<.001), cardiovascular (69 to 15, −78.3%, P<.001), collision (1117 to 416, −62.8%, P<.001), dizziness (865 to 335, −61.3%, <.001), edema (501 to 193, −61.5%, <.001), head trauma (248 to 95, −61.7%, <.001), isolated musculoskeletal trauma (961 to 347, −63.9%, P<.001), skin/nail/hair (587 to 175, −70.2%, P<.001) as well as surgical wound (319 to 94, −70.5%, P<.001).

Clinical conditions related to pulmonary complaints and ILI increased during the comparison periods: fever (640 to 1253, 95.8%, P<.001), lower respiratory infectious symptoms (609 to 1260, 106.9%, P<.001), shortness of breath (2504 to 2726, 8.9%, P=.002) and upper respiratory infectious symptoms (827 to 1825, 120.7%, P<.001) (Table 2).

Year-over-year comparisons of time-sensitive illness based on ICD-10 codes ranged from a decrease of 11.9% (P=0.53) for Acute Cholecystitis, to a drop of 61.4% (P<.001) for Deep Vein Thrombosis (DVT). The diagnosis of Acute Myocardial Infarction (MI) including Acute Coronary Syndrome (ACS), ST-elevation MI, and Non-ST elevation MI) decreased 33% (157 to 105, P=.002). Diagnoses of Cardiac Arrest decreased 39.0% (59 to 36, P=0.02), Gastrointestinal Bleeding by 38.3% (290 to 179, P<.001), all stroke syndromes (hemorrhagic and ischemic) by 17.6% (284 to 234, P=0.03), Pulmonary Embolism (PE) decreased 18.3% (115 to 94, P=0.17), Appendicitis by 15.1% percent (126 to 107, P=0.24) and Seizures diagnoses by 22.0% (41 to 32, P=0.35) (Table 3).

## DISCUSSION

Our study underscores the disturbing finding that patients with time-sensitive and critical conditions such as AMI, cardiac arrest, stroke, venous thrombotic events, and GI bleeding failed to seek emergency medical care during the period of time when public health mitigation measures were in force in Maryland and D.C. While others have highlighted a few specific conditions and general disease categories, our study included all patient clinical presentations and focused on year-over-year trends of a number of the most common time-sensitive illnesses.^3^,^5^,^7^–^9^ The rapid onset of the Covid-19 pandemic caused hospital emergency department patient volumes to plummet throughout the nation, and this trend was evident in the Maryland and Washington, D.C. metro area as well.^3^ Others have provided general evidence of increased morbidity and mortality not attributable to Covid-19, including out of hospital arrest.^4^–^9^ Based on our results, it appears likely that these previous observations were not isolated occurrences.

During the month of March, 2020, public health emergencies were declared in both Maryland and D.C., and executive stay-at-home orders closing all schools and non-essential businesses were put in place.^11^–^13^ Declines in ED patient volumes were subsequently seen across all age groups and genders, with the greatest decline among those 75+. Some of this decrease likely reflected public awareness of reports of increased morbidity and mortality with increasing age.^21^ Additionally, in Maryland, a Johns Hopkins disaster response program called Go Team partnered with the National Guard, Maryland Department of Health, and the University of Maryland to provide stabilizing care to COVID-19 infected nursing home patients in situ which resulted in a reduction in the number of residents who required transport to local EDs for treatment. While patient volumes fell across most racial and ethnic categories, there was no decrease seen in Hispanic or Latinx visits presenting to JHHS EDs. This is not entirely surprising since Hispanic communities in the US and our region have been found to suffer disproportionately higher rates of COVID-19 infection. Despite significant barriers to healthcare access, low rates of medical insurance, and reluctance to seek care, it should be expected that many in this community would turn to emergency care when symptomatic with a possible COVID-19 infection.^22^–^24^

Corresponding to an overall volume decline, was a decrease in most clinical conditions presenting to emergency departments. The exceptions to these downward trends were increased presentations of conditions likely related to COVID-19 such as fever, shortness of breath, and respiratory infections. These complaints, which are potentially indicative of COVID-19 infection, essentially doubled during our study, further accentuating the profound decrease in virtually all other conditions. Our most worrisome finding, however, relates to the significant declines in time-sensitive disease diagnoses. Other researchers have noted similar findings and, indeed, there may be some reasonable explanations for reductions in certain, potentially life-threatening ED presentations.^3^,^7^–^10^,^25^ For instance, patients in isolated settings may not be exerting themselves or confronting significant stressors and, therefore, incidence of acute cardiac events may have decreased. Additionally, studies have demonstrated that people can survive undiagnosed PEs, and there is even some evidence to suggest that conditions as serious as acute appendicitis are over-treated with surgical intervention.^26^–^29^ Taken together, these explanations may elucidate a portion of the decrease in ED volumes of life-threatening conditions. Yet, such possibilities could not reasonably account for the reductions across the numerous time-sensitive illnesses noted in this study.

A more likely explanation is that people suffered serious medical crises and failed to seek appropriate care. A recent article noted that emergency medical services (EMS) in Lodi, CA reported a 45% increase in field cardiac arrest calls, and patients with strokes were arriving too late to receive tissue plasminogen activator (tPA).^4^ Even serious, COVID-19 related complications may have presented to EDs too late for lifesaving care, or patients may have died at home. In Italy, for instance, it was found that a significant percentage of patients who had out-of-hospital cardiac arrests, were also COVID-19+.^9^ Researchers looking at data from the initial COVID-19 outbreak in China, observed that the inflammatory response to the virus can lead to increased rates of thrombosis.^30^ This COVID-19 induced coagulopathy has likely resulted in acute myocardial infarctions, pulmonary embolisms and strokes that did not make it to an ED.

It is highly probable that public health mitigation measures substantially reduced conditions and behaviors that often result in ED visits for occupational injuries, motor vehicle collisions, non-violent trauma, and complications from elective surgeries.^31^ What is more, the expansion of telemedicine services during the pandemic may have provided opportunities for ready access to medical care that previously resulted in ED visits.^32^ Fear, however, likely had the greatest impact on patients failing to seek emergency care. It has been observed anecdotally that anxiety about contracting the Covid-19 infection has caused a significant number of patients to delay or avoid seeking medical care.^4^,^29^,^33^ What our study has clarified is the extent to which ED patients have not sought emergency treatment for time-sensitive, potentially-fatal, medical conditions during the Covid-19 pandemic.

## LIMITATIONS

There are several limitations to our study. First, although the data included all adult patients presenting to our regional hospitals during the prescribed time periods, as with all clinical studies, some data misclassification may have occurred. Second, data from other health systems in the State of Maryland were not analyzed and, therefore, the results of this study may not be generalizable across the state or region. While there was wide geographic distribution amongst the study sites, all hospitals were located within relatively populous areas, the Eastern Shore and Western Maryland may have had different experiences.

## CONCLUSION

ED visits in our health system by patients with time-sensitive conditions that should not have been influenced by the pandemic or public health orders, decreased substantially compared to a previous similar time period. We experienced a significant decline in volumes despite doubling of presentations consistent with Covid-19 symptoms. The reasons are likely multifactorial including: public health stay-at-home orders, closure of non-essential businesses and schools, discontinuation of non-emergent surgical procedures, availability of alternative care options and, perhaps the highest contributor, the generalized fear about contracting the illness.

Hospitals and public health officials need to find a way to better communicate the serious implications of refusing or avoiding emergency medical care. EDs are safe, certainly safer than congregant locations and general indoor public venues. Until the misperception of the risks associated with seeking care at hospital emergency departments are addressed, it is likely that preventable morbidity and mortality will remain a problem.

## Figures and Tables

**Table 1:** Patient demographics by age, gender, race, and ethnicity for time period, March 16 to May 15; 2019 compared with 2020.

	03/16/19 to 05/15/19	03/16/20 to 05/15/20	Change in patient visits, 2019 to 2020		P -Value
	N =	N =	N =	(% =) *	
Total	43,088	27,293	−15,795	(−36.7%)	<.001
Age					
15–24	4,233	2,395	−1,838	(−43.4%)	<.001
25–34	7,705	4,970	−2,735	(−35.5%)	<.001
35–44	6,552	4,525	−2,027	(−30.9%)	<.001
45–54	6,607	4,327	−2,280	(−34.5%)	<.001
55–64	6,691	4,508	−2,183	(−32.6%)	<.001
65–74	5,035	3,050	−1,985	(−39.4%)	<.001
75+	6,246	3,498	−2,748	(−44%)	<.001
Unknown	19	20	1	(5.3%)	1.00
Sex					
Male	19,917	13,814	−6,103	(−30.6%)	<.001
Female	23,164	13,462	−9,702	(−41.9%)	<.001
Other or not specified	7	17	10	(142.9%)	0.06
Race (self-identified)					
American Indian or Alaska Native	60	34	−26	(−43.3%)	0.01
Asian	1,492	930	−562	(−37.7%)	<.001
Black or African American	16,889	10,918	−5,971	(−35.4%)	<.001
Native Hawaiian or Pacific Islander	27	28	1	(3.7%)	1.00
White or Caucasian	19,562	10,989	−8,573	(−43.8%)	<.001
Two or more races	699	485	−214	(−30.6%)	<.001
Other or not specified	4,359	3,909	−450	(−10.3%)	<.001
Ethnicity (self-identified)					
Hispanic or Latino	3,253	3,275	22	(0.7%)	0.79
Not Hispanic or Latino	39,235	23,693	−15,542	(−39.6%)	<.001
Other or not specified	600	325	−275	(−45.8%)	<.001

* Represents change in percent within column category.

**Table 2:** Patients’ chief complaints for time period, March 16 – May 15; 2019 compared with 2020.

	03/16/19 – 05/15/19	03/16/20 – 05/15/20	Change in volume, 2019 to 2020	P-value
	N =	N =	Percent change =	
Abdominal pain	4,742	2,512	−47.0%	<.001
Abnormal finding	456	271	−40.6%	<.001
Abscess	250	110	−56.0%	<.001
Allergic	311	108	−65.3%	<.001
Altered mental status	628	508	−19.1%	<.001
Arrest (cardiac and/or respiratory)	78	51	−34.6%	0.02
Back pain	1,192	435	−63.5%	<.001
Blunt trauma	2,525	1,399	−44.6%	<.001
Burn	144	91	−36.8%	<.001
Cardiovascular (general)	69	15	−78.3%	<.001
Chest pain	3,102	2,042	−34.2%	<.001
Collision	1,117	416	−62.8%	<.001
Constitutional symptoms	264	169	−36.0%	<.001
Dental	352	176	−50.0%	<.001
Device	224	129	−42.4%	<.001
Dizziness	865	335	−61.3%	<.001
Dysrhythmia	526	283	−46.2%	<.001
Edema	501	193	−61.5%	<.001
Ear, nose and throat symptoms (not epistaxis)	338	109	−67.8%	<.001
Environmental	48	24	−50.0%	0.01
Epistaxis	137	63	−54.0%	<.001
Fever	640	1,253	95.8%	<.001
General	580	399	−31.0%	<.001
Genitourinary	1,415	713	−49.6%	<.001
Gastrointestinal (including bleeding)	459	222	−49.4%	<.001
Glucose, abnormal	281	133	−52.7%	<.001
Head trauma	248	95	−61.7%	<.001
Headache	1,165	542	−53.5%	<.001
Hematologic	29	21	−27.6%	#N/A
Hypertension	383	189	−50.7%	<.001
Hypotension	85	36	−57.6%	<.001
Lower respiratory infectious symptoms	609	1,260	106.9%	<.001
Medication management	159	96	−39.6%	<.001
Musculoskeletal (isolated trauma)	961	347	−63.9%	<.001
Musculoskeletal (non-traumatic)	3,323	1,359	−59.1%	<.001
Neurologic	657	422	−35.8%	<.001
Nausea, vomiting and diarrhea	1,244	630	−49.4%	<.001
Ophthalmologic	754	343	−54.5%	<.001
Penetrating trauma	70	75	7.1%	0.74
Pregnancy-related	447	214	−52.1%	<.001
Psychiatric	2,052	1,295	−36.9%	<.001
Referral	92	66	−28.3%	0.05
Seizures	375	247	−34.1%	<.001
Shortness of breath	2,504	2,726	8.9%	0.002
Sickle cell	196	103	−47.4%	<.001
Skin, nails and hair	587	175	−70.2%	<.001
Social issues	123	81	−34.1%	0.00
Substance abuse	1,170	674	−42.4%	<.001
Syncope	591	308	−47.9%	<.001
Upper respiratory infectious symptoms	827	1,825	120.7%	<.001
Weakness	830	537	−35.3%	<.001
Wound	677	343	−49.3%	<.001
Wound check	404	263	−34.9%	<.001
Wound surgery	319	94	−70.5%	<.001
Blank, null, or missing (not mapped)	963	768	−20.2%	<.001
TOTAL	43, 088	27, 293	−36.7%	<.001

**Table 3:** Comparison of emergency department visits for severe illness across Johns Hopkins Health System for time period, March 16 to May 15; 2019 compared with 2020.

	ICD10 Code	03/16/19 to 05/15/19	03/16/20 to 05/15/20	Change in patient visits, 2019 to 2020		P-Value
		N =	N =	N =	(% =)*	
Acute myocardial infarction (MI)						
ST- elevation MI	I21.02–I21.3	48	33	−15	(−31.3%)	0.12
Non-ST-elevation MI	I21.4	89	59	−30	(−33.7%)	0.02
Acute coronary syndrome	I21.9, I24.9	20	13	−7	(−35%)	0.30
Total acute for MI		157	105	−52	(−33.1%)	0.002
Cardiac arrest	I46.8–I46.9	59	36	−23	(−39%)	0.02
Stroke						
Hemorrhagic	I60.0–I62.9	83	70	−13	(−15.7%)	0.33
Ischemic	I63.0–I63.9	201	164	−37	(−18.4%)	0.06
TOTAL FOR STROKE		284	234	−50	(−17.6%)	0.03
Appendicitis	K35–K37	126	107	−19	(−15.1%)	0.24
Venous thromboembolism						
Deep venous thrombosis	I82.4–I82.6	101	39	−62	(−61.4%)	<.001
Pulmonary embolism	I26	115	94	−21	(−18.3%)	0.17
TOTAL FOR VTE		216	133	−83	(−38.4%)	<.001
Acute cholecystitis	K81	67	59	−8	(−11.9%)	0.53
Seizures	G40	41	32	−9	(−22%)	0.35
Gastrointestinal bleed	K92	290	179	−111	(−38.3%)	<.001

* Represents change in percent within column category.
